# Editorial: Sustainable biorefinery/bioprocessing design for functional ingredient production from food waste and byproducts

**DOI:** 10.3389/fnut.2023.1140518

**Published:** 2023-01-20

**Authors:** Qing Jin, Haibo Huang, Yiming Feng

**Affiliations:** ^1^School of Food and Agriculture, University of Maine, Orono, ME, United States; ^2^Department of Food Science and Technology, Virginia Tech, Blacksburg, VA, United States; ^3^Department of Food Science & Nutrition, California Polytechnic State University, San Luis Obispo, CA, United States

**Keywords:** food waste, food byproducts, processing, biorefinery, functional ingredient

Food waste is a global problem that poses severe environmental, economic, and social issues. About one-third of food is lost or wasted globally, accounting for roughly 44% of total solid waste generated worldwide (1, 2). On the other hand, there is an increasing global food demand by 60 to 110% between 2005 and 2050 due to population growth and diet shifts (3, 4). The staggering food waste threatens food security, public health, and our environment. Therefore, it is urgent to seek effective food waste management/utilization strategies to improve environmental and food supply sustainability.

This Research Topic aims to provide the most up-to-date findings on food waste/byproduct characterizations and innovative processing design for functional ingredient generation with the ultimate goal of human health improvement. Under this theme, we have brought together the findings of four papers (two reviews and two research papers) by 23 authors. The food waste/byproducts covered include pomegranate peels, seafood processing side streams, yak bone, and olive leaves.

In a mini review written by Mo et al., the authors introduced the bioactive compounds in pomegranate peel and their functionalities. Pomegranate peel is a by-product generated during pomegranate juice production. It contains bioactive compounds including tannins, flavonoids, phenolic acids, dietary fiber, alkaloids, minerals, and vitamins. These bioactive compounds showed antioxidant, anti-cancer, anti-inflammatory, anti-bacterial activities, and improvement of cardiovascular diseases. All of these functionalities lead to the potential application of pomegranate peel as a natural functional food ingredient for auxiliary treatment of related human diseases. On the other hand, the clinical evidence for the benefits of pomegranate peel bioactive compounds is still needed.

In another review written by Venugopal and Sasidharan, the authors summarized the production of protein from seafood processing discards (e.g., heads, skin, entrails, skeletal frames, and viscera) and effluents. The seafood side streams are good source of protein. For seafood discards, methods including physical and chemical treatments (e.g., green solvent-, ultrasound-, high hydrostatic pressure-, and pulse electric field-assisted extraction), enzymatic hydrolysis, and microbial fermentation were studied for protein generation. As to seafood effluents, they could be used in algae-assisted bioconversion for single cell protein production or processed through flocculation and membrane filtration for protein recovery. Zero-waste biorefinery approaches and the associated challenges were also discussed. The seafood recovered proteins showed several interesting bioactivities and had promising applications in the food industry.

An interesting research paper contributed by Chen et al., the authors investigated three types of yak bone collagen peptides in osteoblasts proliferation. Bone gotten from yak is rich in collagen and minerals and has been traditionally used as Tibetan medicine. In the study, the authors explored three types of di/tripeptides from yak bone. Through *in silico* screening, one type of peptide (MGF) showed excellent MEC3T3-E1 cells' proliferation-promoting ability, suggesting MGF could be used as a potential anti-osteoporosis drug. However, *in vivo* experiment is still needed to validate the current results.

In another interesting paper led by Zhang et al., the authors investigated the phytochemical profiles and biological activities of dried olive leaves, which are processing byproducts of the olive industry. High amounts of flavonoids, iridoids, and triterpenic acids were identified and quantified. Compared with hot air-dried olive leaves, freeze-dried leaves had higher amount of flavonoid aglycone and hydroxytyrosol, and moreover, showed higher radical scavenging, α-glucosidase, α-amylase, and angiotensin-converting enzyme inhibition abilities. A positive correlation was found between olive leave bioactive compounds and biological activities, providing information for value-added products generation from olive leaves.

In conclusion, this themed collection covered a broad scope of Research Topics in functional ingredient production from food waste and byproducts, with unique focuses and insightful views on human health, processing efficiency, and biochemical mechanisms. The new insights highlighted in the collection provide useful information for the potential utilization of certain food waste and food processing byproducts.

## Author contributions

QJ drafted the editorial. HH and YF contributed to editing. All authors conceived and designed the work and approved it for publication.
